# First identification and characterisation of *Brachyspira hyodysenteriae* in pigs in Hong Kong

**DOI:** 10.1186/s40813-019-0133-x

**Published:** 2019-12-04

**Authors:** Kittitat Lugsomya, Friederike Zeeh, Tom La, Nyree Phillips, David J. Hampson

**Affiliations:** 1 Department of Infectious Diseases and Public Health, Jockey Club College of Veterinary Medicine and Life Sciences, City University of Hong Kong, Kowloon Tong Hong Kong, Hong Kong SAR; 2 School of Veterinary and Life Sciences, Murdoch University, Murdoch, Western Australia 6150 Australia

**Keywords:** Swine dysentery, Multilocus sequence typing, Epidemiology, Antimicrobial susceptibility, Minimum inhibitory concentrations, *Brachyspira pilosicoli*, Spirochaetes

## Abstract

Swine dysentery (SD) is an important endemic disease of pigs throughout the world. The most common aetiological agent is the anaerobic intestinal spirochaete *Brachyspira hyodysenteriae.* The related spirochaete *Brachyspira pilosicoli* causes a milder form of colitis. We report the first isolation of *B. hyodysenteriae* and *B. pilosicoli* from a pig farm in Hong Kong. Faecal samples containing mucus or fresh blood were collected from the ground where finisher pigs had just been loaded into a truck for transport to the abattoir. The samples were subjected to selective anaerobic culture and PCR for *B. hyodysenteriae* and *B. pilosicoli*, and two isolates of both species were obtained. The *B. hyodysenteriae* isolates showed clinical resistance to tylosin and lincomycin, whilst the *B. pilosicoli* isolates were resistant to tylosin and showed intermediate susceptibility to lincomycin. The *B. hyodysenteriae* isolates were subjected to multilocus sequence typing and a single previously undescribed sequence type (ST250) was identified. Disease was not recorded in other pigs on the farm, but it may have been masked by the use of antimicrobials. Further work is required to examine the distribution of these two pathogens in this and other farms in Hong Kong and in adjoining mainland China.

Swine dysentery (SD) is an infectious mucohaemorrhagic colitis of pigs which mainly occurs in the grower and finisher phases of production. Uncontrolled SD can cause a severe economic impact through reduced growth rates, mortalities and disruption of pig trade [1]. The most common and widespread aetiological agent of SD is the anaerobic intestinal spirochaete *Brachyspira hyodysenteriae,* although in some regions the related species *Brachyspira hampsonii* and *Brachyspira suanatina* also may cause SD [2]. *Brachyspira pilosicoli* is another species that can cause a milder form of colitis in pigs and in other species [3]. In Hong Kong Special Administrative Region of the People’s Republic of China there are 43 small pig farms with a median of about 200 sows. To date there have been no scientific reports of SD in these farms. This is of interest because of the proximity to the huge swine population in adjoining mainland China, and the reports of SD caused by *B. hyodysenteriae* there and in Taiwan [4, 5]. Hong Kong pig farmers have imported replacement pigs from both countries. In this short communication, we describe the isolation and characterisation of *B. hyodysenteriae* and *B. pilosicoli* from pig faeces on a farm in Hong Kong.

A faecal sample containing mucus and another with a spot of fresh blood were observed on the ground in a pig farm in the New Territories of Hong Kong at a site where fattening pigs had just been loaded into a truck for transportation to the abattoir. As the farmer was concerned about the appearance of the faeces, he collected these and samples from seven other healthy looking finisher pigs. Two months later the farmer collected faecal samples from another 20 finisher pigs, although none of these samples had an abnormal appearance.

The samples were sent to the Veterinary Diagnostic Laboratory at City University of Hong Kong. They were subjected to anaerobic culture for *Brachyspira* species using selective Trypticase Soy Agar (TSA) containing 5% defibrinated ovine blood, 400 μg/ml of spectinomycin and 25 μg/ml each of colistin and vancomycin (Sigma–Aldrich) [6]. The plates were incubated at 37 °C for 5 days in an anaerobic atmosphere generated using the Anaergen™ system (Oxoid). Zones of haemolysis around the inoculated area indicated growth, and confirmation that spirochaetes were present was obtained by re-suspending surface growth and subjecting it to Gram staining before examining at 1000 times magnification. Areas of spirochaetal growth were sub-cultured on selective TSA and then transferred to TSA not containing antimicrobials to grow the purified spirochaetes for further examination. For species identification, the surface growth was subjected to a duplex PCR that is specific for *B. hyodysenteriae* and *B. pilosicoli* [7].

The two initial samples from the floor of the loading area both yielded strongly haemolytic and weakly haemolytic spirochaetal growth on selective TSA. Spirochaetes were not cultured from the other seven samples taken from fattening pigs at that time, or from the 20 samples that were collected 2 months later. Using the species-specific PCR, the strongly haemolytic growth was identified as *B. hyodysenteriae* and the weakly haemolytic growth as *B. pilosicoli.* Two isolates that were identified as *B. hyodysenteriae* were designated as C4B9 and C5B2 and two identified as *B. pilosicoli* were named C4B3 and C5B8.

High molecular weight DNA was extracted from the *B. hyodysenteriae* isolates using the DNeasy Blood and Tissue Kit (Qiagen) according to the manufacturer’s instructions. Multilocus sequence typing (MLST) was conducted using the seven loci used for in the *Brachyspira* MLST scheme [8, 9]. The sequences were submitted to the PubMLST site (https://pubmlst.org/brachyspira/), and sequence types (STs) were assigned within the *B. hyodysenteriae* species-specific MLST scheme.

Using MLST, both *B. hyodysenteriae* isolates were allocated to sequence type 250 (ST250). This is a newly described ST which was defined as singleton. Its nearest neighbour is ST22 with which it shares 4 of 7 loci, and which contains Australian isolates from the 1980s. MLST was not conducted on the *B. pilosicoli* isolates because the cultures were lost following storage.

The susceptibility of the isolates and control strains to tylosin, lincomycin and tiamulin was tested by agar dilution following Clinical and Laboratory Standards Institute (CSLI) guidelines [10]. Control strains were *B. hyodysenteriae* strains B78^T^ and B204, and *B. pilosicoli* strains 95/1000 and WesB. Minimum inhibitory concentrations (MICs) of the isolates and control strains to the three antimicrobial agents were recorded as the lowest concentration which inhibited growth of the bacteria on the plates. The susceptibility of the isolates was interpreted following published breakpoints for establishing clinical resistance in agar dilution testing in *Brachyspira* species. For tiamulin, the CSLI breakpoint of > 0.25 μg/ml was used [10]. For tylosin and lincomycin, MICs of > 16 μg/ml and > 1 μg/ml were used, respectively. These values followed Rønne and Szancer’s criteria [11], as CSLI breakpoints for these two antimicrobials are not available for *Brachyspira* species. The MIC values of the Hong Kong isolates and the control strains of both species are shown in Table 1. All the Hong Kong strains were recorded as being susceptible to tiamulin and resistant to tylosin. Furthermore, the *B. hyodysenteriae* isolates were resistant to lincomycin, but the *B. pilosicoli* isolates showed intermediate susceptibility.

**Table 1:** Sequence types and their interpreted susceptibility and minimum inhibitory concentrations of the eight *Brachyspira* isolates/strains

Isolate/strain	Species-specific sequence type	Susceptibility (MIC in μg/ml)		
		Tylosin	Lincomycin	Tiamulin
*B. hyodysenteriae* C4B8	250	R (> 16)	R (> 72)	S (< 0.063)
*B. hyodysenteriae* C5B9	250	R (> 16)	R (> 72)	S (< 0.063)
*B. hyodysenteriae* B78^T^	56	I (4)	S (< 2)	S (< 0.063)
*B. hyodysenteriae* B204	54	R (> 16)	I (36)	S (< 0.063)
*B. pilosicoli* C4B3	NK	R (> 16)	I (16)	S (< 0.063)
*B. pilosicoli* C4B8	NK	R (> 16)	I (16)	S (< 0.063)
*B. pilosicoli* 95/1000	82	S (< 0.5)	I (36)	S (< 0.063)
*B. pilosicoli* WesB	68	S (< 0.5)	S (< 2)	S (< 0.063)

The isolates C4B8, C5B9, C4B3 and C4B8 originated from a pig herd in Hong Kong Special Administrative Region. Minimum inhibitory concentrations (MIC) were determined by agar dilution and interpreted according to [11] for tylosin, lincomycin and to [10] for tiamulin.

*NK* not known, *S* susceptible, *I* intermediate, *R* resistant

This is the first identification of *B. hyodysenteriae* and of *B. pilosicoli* in pigs in Hong Kong. The single ST of *B. hyodysenteriae* suggested that only one strain might be present on the farm. From MLST data, this strain was different from strains previously identified in other parts of the world. Due to the limited number of analysed isolates, the meaning of this finding is unclear. Either this *B. hyodysenteriae* has been introduced into Hong Kong through importation of live pigs, or there might be a local population of *Brachyspira*. Here, the original source of infection remains unclear.

Spirochaetes were not isolated from other pigs on the farm, and there was no sign of disease other than the two atypical faecal samples derived from finisher pigs. On subsequent discussion, the farmer revealed that he injected any pigs with diarrhoea using a quinoxaline-N,N-dioxide antibacterial agent. Earlier he had been injecting pigs with a combination of lincomycin and spectinomycin (concentration and dosage unknown). Tiamulin had not been used on the farm. The farmer sourced his antimicrobials directly from mainland China. These comments suggested that although disease was occasionally seen it was being masked by the use of antimicrobials. The isolates showed elevated MICs to tylosin and to lincomycin in the case of *B. hyodysenteriae*, but not to tiamulin, and this reflected the antimicrobial usage on the farm.

High MICs to all three antimicrobials have been recorded in *B. hyodysenteriae* and *B. pilosicoli* isolates in nearby Taiwan, and attributed to frequent use of these antimicrobials in pig production there [5].

Further work is required to assess the extent of infection in the farm from which the isolates were recovered, as well as to determine whether infection with pathogenic *Brachyspira* species occurs in other pig farms in Hong Kong. Both species also can infect chickens [12, 13], so examination of layer flocks in Hong Kong for infection also is warranted. Furthermore, more *B. hyodysenteriae* isolates should be characterised for better understanding of their epidemiology and control measures of SD.

## Data Availability

Not applicable.

## Abbreviations

Hong Kong SAR: Hong Kong Special Administrative Region
MIC: Minimum inhibitory concentrations
MLST: Multilocus sequence typing
PCR: Polymerase chain reaction
SD: Swine dysentery
ST: Sequence type
TSA: Trypticase Soy Agar
